# New Process Combining Fe-Based Chemical Looping and Biomass Pyrolysis for Cogeneration of Hydrogen, Biochar, Bio-Oil and Electricity with In-Suit CO_2_ Separation

**DOI:** 10.3390/molecules28062793

**Published:** 2023-03-20

**Authors:** Xing Zhou, Huilong Jin, Na Li, Xiaolong Ma, Zichuan Ma, Pei Lu, Xiaomeng Yao, Shenna Chen

**Affiliations:** 1 College of Engineering, Hebei Normal University, Shijiazhuang 050024, China; zhoux@hebtu.edu.cn (X.Z.);; 2 Hebei Key Laboratory of Inorganic Nano-Materials, College of Chemistry and Materials Science, Hebei Normal University, Shijiazhuang 050024, China; 3 Inner Mongolia Key Laboratory of High-Value Functional Utilization of Low Rank Carbon Resources, College of Chemical Engineering, Inner Mongolia University of Technology, Huhhot 010051, China; 4 School of Environmental Science and Engineering, Hebei University of Science and Technology, Shijiazhuang 050018, China; 5 Hebei Key Laboratory of Environmental Change and Ecological Construction, School of Geographical Sciences, Hebei Normal University, Shijiazhuang 050024, China

**Keywords:** hydrogen, chemical looping, cogeneration, exergy efficiency, performance analysis

## Abstract

Fe-based chemical looping gasification is a clean biomass technology, which has the advantage of reducing CO_2_ emissions and the potential of self-sustaining operation without supplemental heating. A novel process combining Fe-based chemical looping and biomass pyrolysis was proposed and simulated using Aspen Plus. The biomass was first subjected to pyrolysis to coproduce biochar, bio-oil and pyrolysis gas; the pyrolysis gas was subjected to an Fe looping process to obtain high-purity hydrogen and carbon dioxide. The influences of the pyrolysis reactor operating temperature and fuel reactor operation temperature, and the steam reactor and air reactor on the process performance are researched. The results showed that, under the operating condition of the established process, 23.07 kg/h of bio-oil, 24.18 kg/h of biochar, 3.35 kg/h of hydrogen and a net electricity of 3 kW can be generated from 100 kg/h of rice straw, and the outlet CO_2_ concentration of the fuel reactor was as high as 80%. Moreover, the whole exergy efficiency and total exergy loss of the proposed process was 58.98% and 221 kW, respectively. Additionally, compared to biomass direct chemical looping hydrogen generation technology, the new process in this paper, using biomass pyrolysis gas as a reactant in the chemical looping hydrogen generation process, can enhance the efficiency of hydrogen generation.

## 1. Introduction

Hydrogen is regarded as one of the most promising energy sources due to its high energy density and cleanness [1,2]. Nevertheless, the H_2_ molecule does not exist in nature, and most of the hydrogen exists in water [3,4]. Nowadays, natural gas steam reforming [5] is the most extensive hydrogen production method, which accounts for 48% of the world’s hydrogen production [6,7]. Nevertheless, natural gas steam reforming needs H_2_ separation and purification as well as the production of a large amount of carbon dioxide (CO2) [8]. Accordingly, it is necessary to develop alternative and renewable sources to produce H_2_ [9].

Biomass is considered to be a promising renewable source with carbon neutrality to produce H_2_. From the view of thermodynamics, steam gasification of biomass to generate 53–55 vol% of H_2_ should be the most popular method for H_2_ production [6,10], while only 8–10 vol% of H_2_ can be generated by air gasification of biomass [11]. Nevertheless, steam gasification of biomass requires heat because it is an endothermic reaction, whereas air gasification of biomass is an exothermic process due to the partial combustion of biomass with oxygen. Therefore, the mixing of steam with air in biomass hydrogen production has been extensively developed; however, its H_2_ content is only 25–30 vol% [12]. Thus, an H_2_ purification and CO_2_ separation system are also needed.

**Figure 1:**
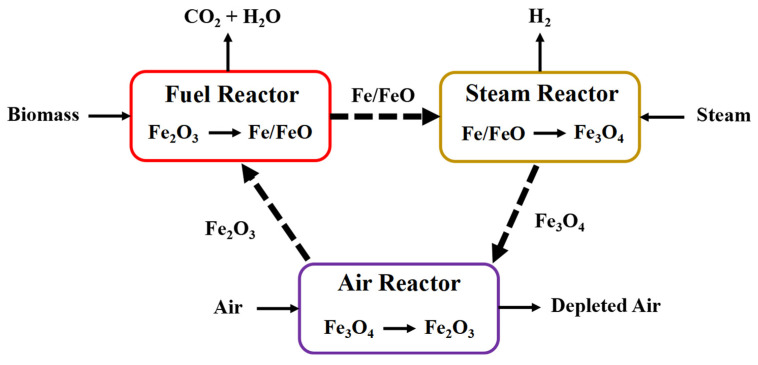
Schematic diagram of biomass direct chemical looping hydrogen generation.

As shown in Figure 1, biomass direct chemical looping is a novel H_2_ generation method; that is, the solid biomass is directly mixed with the oxygen carrier. High-purity H_2_ and sequestration-ready CO_2_ can be generated by biomass direct chemical looping without purification technology, which can theoretically reduce energy consumption [13].

However, there are some disadvantages in biomass direct chemical looping for hydrogen generation technology. Firstly, the reaction rate of solid biomass with solid oxygen carriers is low due to the low efficiency of solid–solid contact [14]. Secondly, it is hard to separate oxygen carriers from biomass ash and/or biochar [15]. Third, bio-oil has certain value: directing bio-oil to reduce oxygen carrying will reduce the economy of the chemical looping system. More importantly, in the fuel reactor, solid biomass, small molecular gas products (CO, CH_4_), biochar, bio-oil and oxygen carrier reactions will interact with each other. For example, biochar will hinder the reduction of pyrolysis gas to oxygen carrier, and tar will affect the pore structure of biochar [16,17]. If a chemical looping is coupled to a polygeneration system, biochar, bio-oil, pure hydrogen and sequestration-ready CO_2_ can be obtained simultaneously. Additionally, the efficiency of the whole biomass conversion process can be increased.

Therefore, it will be interesting to study the combination of chemical looping and polygeneration concepts for biomass to H_2_ systems. Nguyen et al. [18] proposed novel polygeneration concepts to cogenerate H_2_, biochar, CH_4_, bio-oil and methanol from biomass hydropyrolysis and hydrodeoxygenation integrated with water electrolysis. The results demonstrated that the promising processes are those with H_2_ generation, while the risky systems are those with water electrolysis. Situmorang et al. [19] presented a new process of H_2_ generation by integrating steam bio-oil reforming as well as a biochar chemical looping hydrogen production system. The results showed that the proposed system can enhance H_2_ generation efficiency by more than 50% compared with the biomass direct chemical looping hydrogen generation process.

However, to the best of the authors’ knowledge, the polygeneration system for H_2_, biochar, bio-oil and electricity generation and coproduction by biomass pyrolysis coupled with a chemical looping hydrogen generation process using biomass pyrolysis gas as a reactant has not been reported.

In this paper, a new process integrating biomass pyrolysis and chemical looping H_2_ generation using pyrolysis gas from the pyrolysis of biomass as the reactant is designed and simulated to produce H_2_, bio-oil, biochar and electricity. According to the energy and mass balances of the whole process, the exergy efficiency and exergy loss, and the influences of the pyrolysis reactor operating temperature and operation temperatures at the fuel reactor, steam reactor and air reactor on the process performance are studied. It is expected to achieve a novel process to effectively produce H_2_, bio-oil and biochar from biomass.

## 2. Results and Discussion

### 2.1. Material and Energy Calculation of the Proposed System

Based on the simulation results, the whole process can produce 23.07 kg/h of bio-oil (Table 1), 24.18 kg/h of biochar, 52.75 kg/h of pyrolysis gas, 3.35 kg/h of hydrogen and a net electricity of 3 kW per 100 kg/h of rice straw. A total of 100 kg/h of Fe_2_O_3_/Al_2_O_3_ oxygen carrier, which contains 45% excess as heat carrier, is circulated to completely convert pyrolysis gas in the chemical looping hydrogen generation unit.

**Figure 2:**
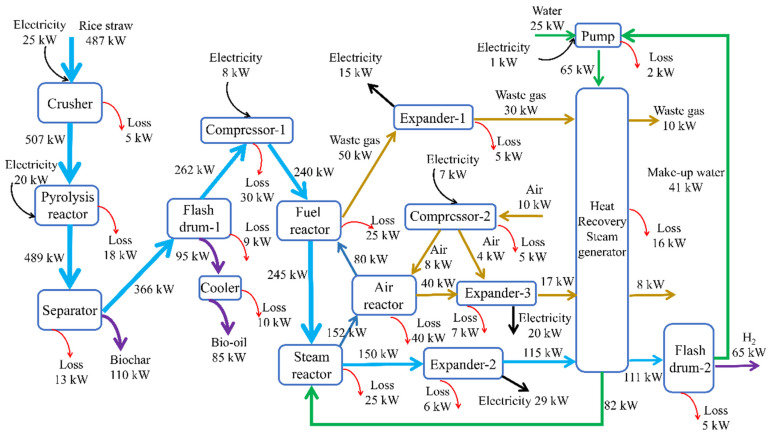
Exergy flowchart of the proposed process.

The steam reactor needed 45 kg/h of water, and about 36.65 kg/h of water was recycled back into the steam reactor, which can be gained from the flash drum. Thus, about 8.35 kg/h of make-up water is needed from outside. Table 2 demonstrates the stream results of the proposed system shown in Figure 2 and Table 3 shows the electricity results of the proposed system.

According to Equations (4)–(6), the efficiency of H_2_ generation, efficiency of bio-oil generation and efficiency of biochar generation are 6.35%, 23.07% and 24.18%, respectively. The efficiency of hydrogen generation from coal can reach 15% [20]; in contrast, that for biomass can only approach about 5% [14]. Nevertheless, although the efficiency of hydrogen generation for pyrolysis gas is lower than coal, using pyrolysis gas as a reductant in the chemical looping hydrogen generation process can enhance the efficiency of hydrogen generation from biomass.

### 2.2. Exergy Flowchart

The exergy flowsheet of the proposed system is demonstrated in Figure 2. It can be seen that the overall exergy efficiency of the proposed process is as high as 58.98%. The exergy loss of the whole system is 221 kW. Additionally, the chemical reactions’ irreversibility is the main reason for the losses of exergy for the pyrolysis reactor, fuel reactor, steam reactor and air reactor. The compression and expansion processes’ irreversibility is the primary reason for the exergy losses of compressor-1, compressor-2, the pump, expander-1, expander-2 and expander-3. Heat loss is the main reason for the exergy loss of the heat recovery steam generator, separator, flash drum-1 and flash drum-2.

In order to improve the proposed system’s exergy efficiency, the fuel reactor, air reactor, steam reactor, heat recovery steam generator and compressor-1 are preferred to be improved. Improving the chemical reaction efficiency is necessary to reduce the loss of exergy for the fuel reactor, air reactor and steam reactor such as improving the performance of the oxygen carrier and enhancing the mass and heat transfer efficiency in the above reactor. Enhancing the mechanical efficiency of the compressor can increase exergy efficiency. Additionally, the heat recovery steam generator’s exergy efficiency can be increased by improving the efficiency of heat utilization.

### 2.3. Effect of Pyrolysis Temperature

**Figure 3:**
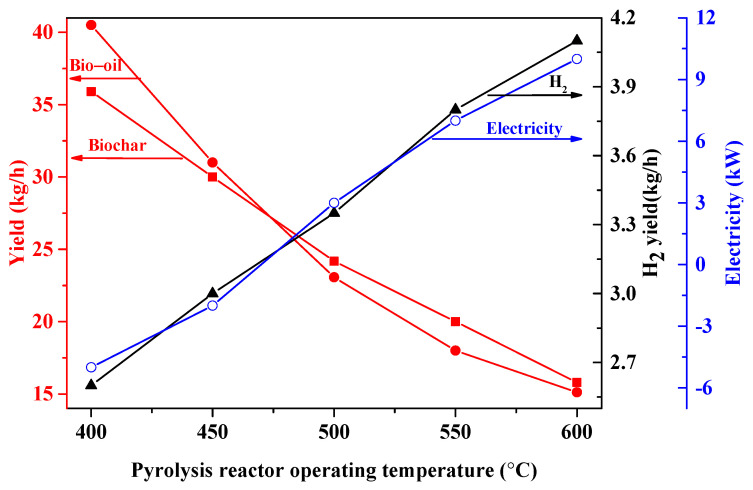
Influence of the pyrolysis reactor operating temperature on hydrogen, biochar, bio-oil and electricity production. The temperature of steam reactor and air reactor are kept at 700 and 980 °C.

The influence of the pyrolysis reactor operating temperature on the hydrogen production yield, bio-oil production yield, biochar production yield and electricity production are shown in Figure 3. The temperatures of the fuel reactor, steam reactor and air reactor are kept at 900 °C, 700 °C and 980 °C, respectively. As the pyrolysis reactor operating temperature increases from 400 °C to 600 °C, the bio-oil production yield and biochar production yield decline from 40.5 kg/h and 35.9 kg/h to 15.12 kg/h and 15.8 kg/h, respectively. The reason is that bio-oil and biochar are converted to pyrolysis gas owing to the primary secondary cracking reactions [10,21].

Nevertheless, the H_2_ production yield and electricity production are enhanced from 2.6 kg/h and −5 kW to 4.1 kg/h and 10 kW, respectively. The reason is that elevating the amount of pyrolysis gas means more Fe_2_O_3_ is converted into FeO and Fe, which react with steam to produce hydrogen. At the same time, more related gas (waste gas from the fuel reactor, H_2_/steam from the steam reactor) is generated, which affects the work of the expanders.

### 2.4. Effect of Fuel Reactor Operation Temperature

**Figure 4:**
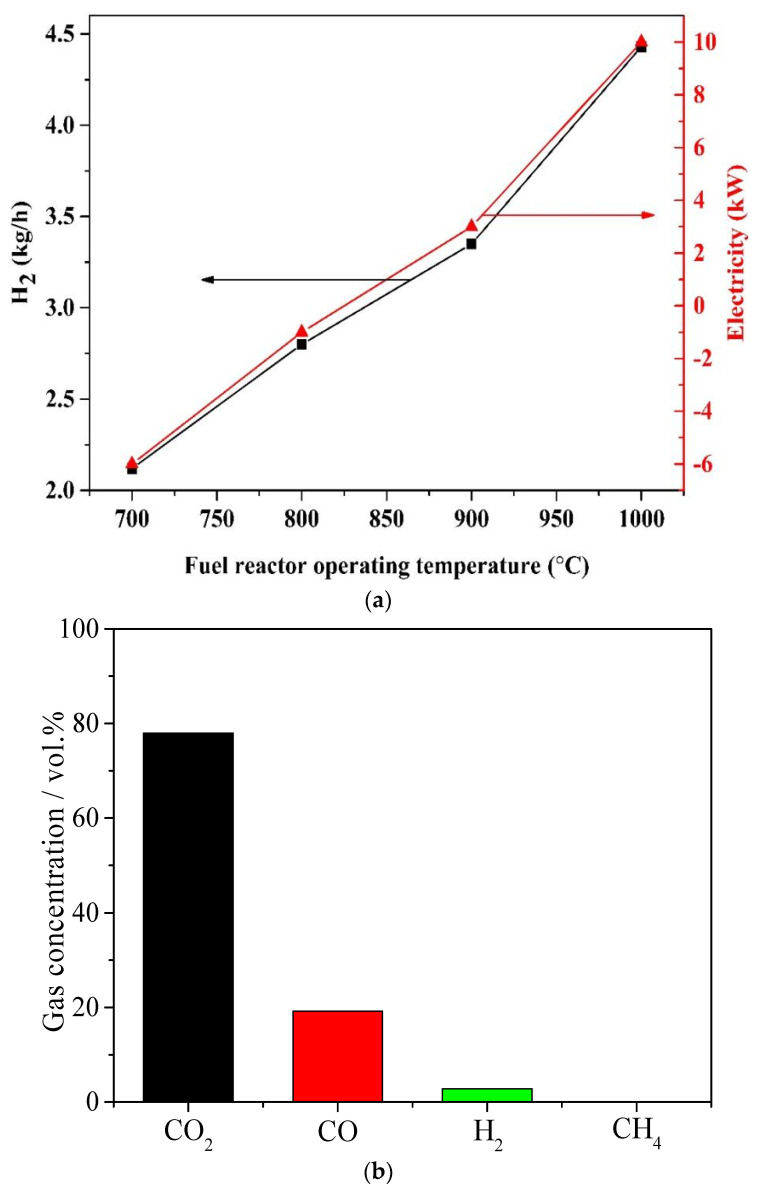
Influence of the fuel reactor operating temperature, the temperatures of pyrolysis reactor, steam reactor and air reactor are 500 °C, 700 °C and 980 °C. (**a**) Hydrogen and electricity production. (**b**) The gas composition of the outlet of fuel reactor at 900 °C.

For the chemical looping process, the fuel reactor operation temperature is usually from 750 °C to 950 °C to convert Fe_2_O_3_ into FeO/Fe. The influence of the fuel reactor operation temperature on the hydrogen production yield and electricity production is demonstrated in Figure 4. The temperatures of the pyrolysis reactor, steam reactor and air reactor are maintained at 500 °C, 700 °C and 980 °C, respectively.

As the fuel reactor operating temperature increases from 700 °C to 1000 °C, the H_2_ production yield and electricity production increase from 2.12 kg/h and 6 kW to 4.43 kg/h and 10 kW, respectively. The pyrolysis gas reaction with Fe_2_O_3_ is an endothermic process, which means more FeO is produced with the increase in the fuel reactor temperature; thus, more H_2_ is produced in the steam reactor. Additionally, more waste gas (CO_2_ and steam) is produced in the fuel reactor, which results in the increased electricity production of expander-1. However, a higher fuel reactor operation temperature could lead to insufficient air reactor heat [22].

Table 4 shows the outlet gas composition of the fuel reactor at 900 °C. It can be seen that the CO_2_ concentration of the dry base is about 80%, indicating that the biomass pyrolysis gas in the fuel reactor has been basically oxidized. Therefore, 900 °C is suitable for the fuel reactor.

### 2.5. Effect of Steam Reactor Temperature

**Figure 5:**
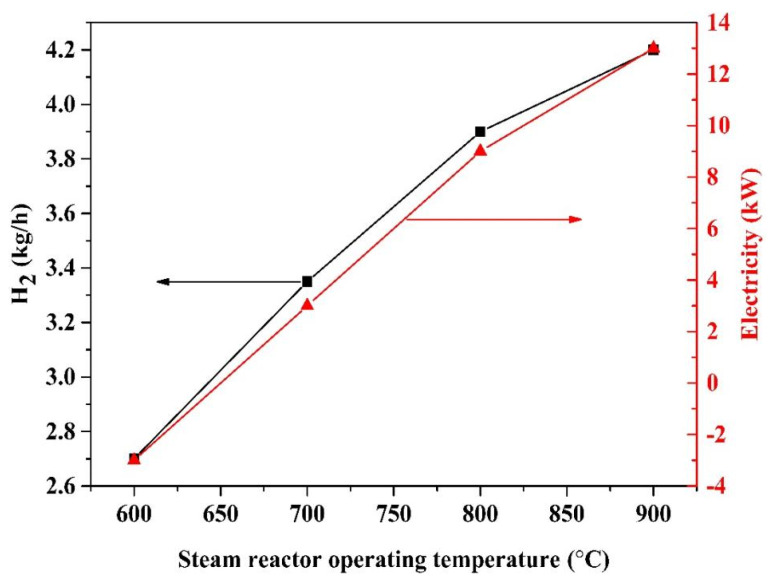
Influence of the steam reactor operating temperature on hydrogen and electricity production, the temperatures of the pyrolysis reactor, fuel reactor and air reactor are 500 °C, 900 °C and 980 °C.

In the steam reactor, H_2_ is generated from steam via the oxidation of FeO/Fe to Fe_3_O_4_. The influence of the steam reactor operating temperature on the H_2_ production yield and electricity production is demonstrated in Figure 5. The temperatures of the pyrolysis reactor, fuel reactor and air reactor are maintained at 500 °C, 900 °C and 980 °C, respectively. As the steam reactor operating temperature increases from 600 °C to 900 °C, the hydrogen production yield decreases from 2.7 kg/h to 4.2 kg/h, whereas the electricity production increases from −3 kW to 13 kW.

Steam with FeO/Fe are exothermic reactions with the equilibrium obtained at low temperatures. The lower the steam reactor operating temperature is, the higher the hydrogen production yield becomes. Nevertheless, the heat is not to support the chemical looping hydrogen generation process auto-thermally at low temperatures. As the steam reactor operating temperature goes up, the conversion of steam decreases, resulting in an increase in expander-2 electricity generation.

### 2.6. Effect of Air Reactor Operating Temperature

**Figure 6:**
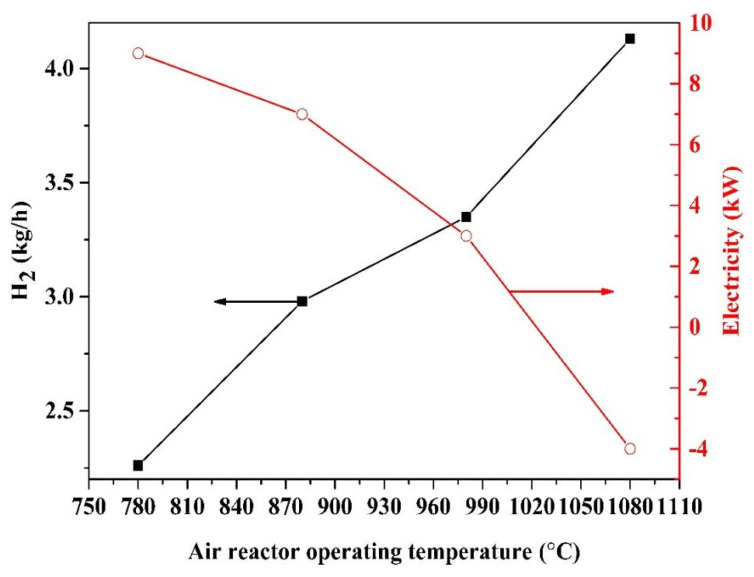
Influence of the air reactor operating temperature on hydrogen and electricity production, the temperatures of the pyrolysis reactor, fuel reactor and steam reactor are 500 °C, 900 °C and 700 °C.

The operating temperature of the air reactor is a crucial parameter for hydrogen and electricity production. The air reactor operating temperature can affect the fuel reactor temperature due to the energy balance for the chemical looping process. On the other hand, the air reactor operating temperature is limited by the oxygen carrier temperature’s endurance. The influence of the air reactor operating temperature on hydrogen and electricity is shown in Figure 6.

The temperatures of the pyrolysis reactor, fuel reactor and steam reactor are kept at 500 °C, 900 °C and 700 °C, respectively. As the air reactor operating temperature increases from 780 °C to 1080 °C, the hydrogen production yield enhances from 2.26 kg/h to 4.13 kg/h, whereas the electricity production drops from 9–4 kW. The reason for the decrease in net electricity is that the air flow declines with the rise in the air reactor operating temperature, leading to the temperature declines of the expander-3 inlet stream.

The increase in the air reactor operation temperature leads to the increase in the Fe_2_O_3_ temperature, and more FeO is generated in the fuel reactor, which results in the increase in H_2_ production in the steam reactor. Nevertheless, the high operating temperature of the reactors can cause the sintering of the oxygen carrier [23]. Therefore, the air reactor operation temperature should be less than 1000 °C.

Table 4 shows the gas composition and oxygen carrier results based on Figure 4, Figure 5 and Figure 6. As expected, under optimal conditions, the dry base CO_2_ concentration at the outlet of the fuel reactor is about 80%. All the Fe is oxidized by steam into FeO in the steam reactor, and the product is pure H_2_.

## 3. Methods and Materials

### 3.1. Materials

China has abundant straw production and the price of straw is low. Therefore, rice straw is selected as the raw material and its properties are listed in Table 5.

### 3.2. Model Description

**Figure 7:**
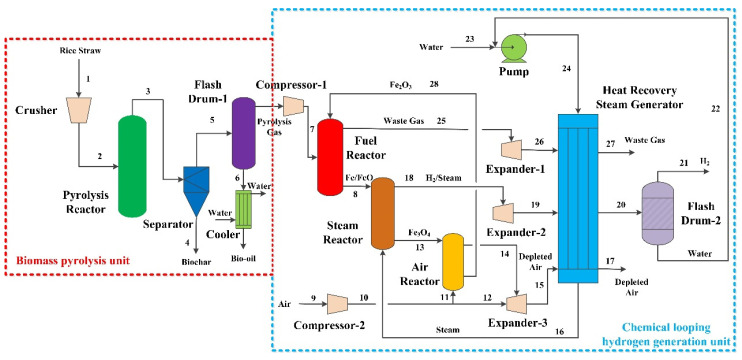
Flowsheet of the process for hydrogen, biochar and bio-oil coproduction by biomass pyrolysis integrated with chemical looping hydrogen generation.

The new process for hydrogen, bio-oil and biochar from biomass was designed in Aspen Plus V9. To simulate the process, all of the reactions are assumed to be in equilibrium and the system is assumed to be in a steady state. As shown in Figure 7, the cogeneration system primarily consists of two units: (i) biomass pyrolysis and (ii) chemical looping hydrogen generation.

#### 3.2.1. Biomass Pyrolysis Unit

First of all, 100 kg/h of rice straw is transported to the crusher to obtain the required particle size. Particle size of rice straw obtained through the crusher less than 1 mm accounts for 30%. Particle size in the range of 1–2 mm accounts for 40% and that of more than 2 mm accounts for 30%. Then, the crushed rice straw is sent to the pyrolysis reactor. According to the related reference [20], the reaction temperature and pressure of the pyrolysis reactor are set to be 600 °C and 0.1 MPa, respectively. Exiting from the pyrolysis reactor, biochar is separated from the mixture of gas and bio-oil via the separator. The mixture of gas and bio-oil is then transported to the flash drum-1 and the bio-oil is obtained. The pyrolysis gas is sent to the chemical looping hydrogen generation unit.

RK-SOAVE is selected as the property method to achieve the mass and energy balances in the biomass pyrolysis unit. Rice straw, biochar and ash are regarded as nonconventional components and their enthalpy is calculated using HCOALGEN and DCOALIGT.

Bio-oil is a complex mixture of hundreds of organic compounds and the description of pyrolysis modeling for bio-oil is difficult [18]. Therefore, the modeling of bio-oil needs to be simplified. Bio-oil is simulated as a mixture of water, hydroxyacetaldehyde, hydroxypropanone, propionic acid, isoeugenol, acetic acid, phenol, syringol and (5*H*)-furan-2-one [21] in this paper. 

Pyrolysis gas is considered to be a mixture of H_2_, CO, CH_4_, CO_2_ and others (C_2_H_6_, C_2_H_4_, etc.). Crusher, SSplit and Flash blocks are used to simulate the crusher, separator and flash drum-1, respectively. RYield, REquil and RGibbs blocks are developed to model the pyrolysis reactor. As shown in Table 6, the simulated results of biomass pyrolysis are verified by the work of Zhang [20]. Due to the difference between simulation and actual reaction process, there are relative errors between simulation results and experiment results. The relative errors between the experiment results and simulation results are small, hence the simulation results are rational.

#### 3.2.2. Chemical Looping Hydrogen Generation Unit

The pyrolysis gas from the biomass pyrolysis unit is compressed to 1 MPa by the compressor-1 and sent to the fuel reactor (900 °C, 1 MPa) and reacts with oxygen carrier Fe_2_O_3_/Al_2_O_3_ (100 kg, mass ratio 4:6), which is chosen as oxygen carrier due to its advantages of low cost, thermodynamic property and oxygen capacity [6], generating H_2_O, CO_2_ and Fe/FeO.

After that, Fe/FeO is sent to the steam reactor (700 °C, 1 MPa) and partially oxidized by steam with the production of H_2_ and Fe_3_O_4_. Then, Fe_3_O_4_ is transported to the air reactor (980 °C, 1 MPa) and completely oxidized to Fe_2_O_3_ by air. The regenerated oxygen carrier is sent to the fuel reactor and a cycle is completed.

The flue gas is utilized in expanders to generate electricity and introduced to the heat recovery steam generation. The operating parameters of the fuel reactor, steam reactor and air reactor are determined based on previous publications [19,22]. The reactions that take place in the chemical looping hydrogen generation unit can be expressed by Equations (1)–(3).

Fuel reactor C_x_H_y_O_z_ (pyrolysis gas) + Fe_2_O_3_ → CO_2_ + H_2_O + FeO/Fe (1)

Steam reactor FeO/Fe + H_2_O → Fe_3_O_4_ + H_2_ (2)

Air reactor 4Fe_3_O_4_ + O_2_ → 6Fe_2_O_3_ (3)

RK-SOAVE is developed for nonpolar or mildly polar mixtures such as hydrocarbons and light gases (e.g., carbon dioxide, hydrogen sulfide and hydrogen). Therefore, RK-SOAVE is selected as the property method in the chemical looping hydrogen generation unit to achieve the mass and energy balances. Fe_2_O_3_, Al_2_O_3_, Fe, FeO and Fe_3_O_4_ are assumed to be solid components. The minimum ΔT for heat exchanger is 10 °C. The isentropic efficiency and mechanical efficiency of the pressure changer (compressor, expander and pump) are 0.80 and 0.90, respectively. RGibbs block is used to model the fuel reactor, steam reactor and air reactor [23]. Flash, Pump and MHeatX blocks are developed to simulate the flash drum-2, pump and heat recovery steam generator, respectively. Compr block is used to model the compressor-1, compressor-2, expander-1, expander-2 and expander-3. The Aspen model and input data for the equipment of the proposed system are given in Table 7.

### 3.3. Evaluation Index of the Proposed System

The performance of the proposed process is assessed by hydrogen production yield, bio-oil production yield, biochar production yield, efficiency of hydrogen generation, the efficiency of bio-oil generation, the efficiency of biochar generation as well as electricity production.

(4) $$H_{2}\;\;\mathrm{generation}\;\;\mathrm{efficiency}=\frac{H_{2}\;\;\mathrm{generation}\;\;\mathrm{yield}}{\mathrm{Mass}\;\;\mathrm{of}\;\;\mathrm{pyrolysis}\;\;\mathrm{gas}}$$

(5) $$\mathrm{Bio}-\mathrm{oil}\;\;\mathrm{generation}\;\;\mathrm{efficiency}=\frac{\mathrm{Bio}-\mathrm{oil}\;\;\mathrm{generation}\;\;\mathrm{yield}}{\mathrm{Mass}\;\;\mathrm{of}\;\;\mathrm{biomass}}$$

(6) $$\mathrm{Biochar}\;\;\mathrm{generation}\;\;\mathrm{efficiency}=\frac{\mathrm{Biochar}\;\;\mathrm{generation}\;\;\mathrm{yield}}{\mathrm{Mass}\;\;\mathrm{of}\;\;\mathrm{biomass}}$$

(7) $$\mathrm{Electricity}\;\;\mathrm{production}=\sum E_{\mathrm{expander}}-\sum E_{\mathrm{compressor}}-\sum E_{\mathrm{pump}}$$

where E_expander_, E_pump_ and E_compressor_ are the electricity produced by the expander and the electricity consumed by the pump and compressor, respectively.

### 3.4. Thermodynamic Analysis

Exergy usually consists of kinetic exergy E^KN^, potential exergy E^PT^, physical exergy E^PH^ and chemical exergy E^CH^ [24]. The kinetic exergy and potential exergy can be neglected in a chemical industrial process [25]. Thus, exergy is defined as:

(8) $${E=E}_{}^{\mathrm{CH}}{+E}_{}^{\mathrm{PH}}$$

The E^CH^ of gas mixture can be expressed as follows [26]:

(9) $$E_{}^{\mathrm{CH}}=\sum x_{i}E_{0,i}^{\mathrm{CH}}{+\mathrm{RT}}_{0}\sum x_{i}{\mathrm{lnx}}_{i}$$

The E^CH^ of nongaseous mixture can be defined as follows [27]:

(10) $$E_{}^{\mathrm{CH}}=\sum x_{i}E_{0,i}^{\mathrm{CH}}$$

where $E_{0,i}^{\mathrm{CH}}$ is the component’s standard E^CH^, x_i_ stands for the i component’s molar fraction, T_0_ is 298.15 K, R equals 8.314 kJkmol^−1^K^−1^ and the subscript 0 stands for 1 atm and 298.15 K (the reference state). The standard chemical exergy values are demonstrated in those references [26,27,28].

The E^PH^ is expressed as follows [12]:

(11) $$E_{}^{\mathrm{PH}}{=(H-H}_{0}{)-T}_{0}{(S-S}_{0})$$

where H and S stand for the enthalpy and entropy at a given temperature and pressure, H_0_ and S_0_ are the enthalpy and entropy at 298.15 K and 1 atm. H, H_0_, S and S_0_ are calculated by Aspen Plus. In addition, the ash exergy and rice straw E^PH^ are neglected owing to minor contribution [29].

Additionally, the exergy of biomass can be expressed as:

(12) $$E_{\mathrm{biomass}}^{}{=\beta \times \mathrm{LHV}}_{\mathrm{biomass}}$$

(13) $$\beta =\frac{1.0414+0.0177[H/C]-0.3328[O/C]\{1+0.0537[H/C]\}}{1-0.4021[O/C]}$$

(14) $${\mathrm{LHV}}_{\mathrm{biomass}}=0.0041868(1+0.15[O])(7837.667[C]+33888.889[H]-[O]/8)$$

where C, H and O are carbon, hydrogen and oxygen elements, and are obtained from the ultimate analysis. In addition, the calculation method of exergy for biochar and bio-oil is listed in reference [29].

The exergy destruction E_D_ and exergy efficiency $\eta_{\mathrm{ex}}$ can be calculated as follows:

(15) $$E_{D}=\sum E_{\mathrm{in}}-\sum E_{\mathrm{out}}$$

(16) $$\eta_{\mathrm{ex}}=\sum E_{\mathrm{out}}/\sum E_{\mathrm{in}}$$

where E_in_ stands for the entering exergy and E_out_ is the outflow exergy.

## 4. Conclusions

A new process for biochar, bio-oil, H_2_ and power cogeneration from rice straw was developed. The whole process can produce 23.07 kg/h of bio-oil, 24.18 kg/h of biochar, 52.75 kg/h of pyrolysis gas and 3.35 kg/h of H_2_ per 100 kg/h of rice straw. The results showed that the process combined biomass pyrolysis with chemical looping hydrogen production, and the hydrogen production efficiency (6.35%) is higher than that of biomass direct chemical looping hydrogen production technology. The proposed process also produces positive net electricity, which is 3 kW per 100 kg/h of rice straw. Additionally, the overall exergy efficiency of the proposed process is 58.98% and the total exergy loss is 221 kW. Furthermore, an economic assessment needs to be carried out to implement this new process.

## Figures and Tables

**Table 1:** Simulation results for the components of the bio-oil.

Components	Value (wt%)
Water	28.11
Hydroxyacetaldehyde	25.32
Hydroxypropanone	14.12
Propionic acid	2.63
Isoeugenol	9.86
Acetic acid	15.37
Phenol	1.52
Syringol	0.75
(5*H*)-furan-2-one	2.32

**Table 2:** Stream and electricity results of the proposed process.

Stream	Component	Mass Flow (kg/h)	P (MPa)	T (°C)
1	Rice straw	100	0.1	25
2	Crushed rice straw	100	0.1	25
3	Biochar, bio-oil and pyrolysis gas	100	0.1	600
4	Biochar	24.18	0.1	600
5	Bio-oil and pyrolysis gas	75.82	0.1	600
6	Bio-oil	23.07	0.1	600
7	Pyrolysis gas	52.75	1	600
8	Fe/FeO	90	1	900
9	Air	120	0.1	25
10	Compressed air	120	1.2	200
11	Compressed air	80	1.2	200
12	Compressed air	40	1.2	200
13	Fe_3_O_4_	95	1	850
14	Depleted air	75	1.1	850
15	Depleted air	115	0.1	350
16	Steam	45	1	250
17	Depleted air	115	0.1	50
18	Steam + H_2_	40	1	850
19	Hot water + H_2_	40	0.1	400
20	Cooled water + H_2_	40	0.1	60
21	H_2_	3.35	0.1	25
22	Recycled water	36.65	0.1	25
23	Make-up water	8.35	0.1	25
24	Total water	45	1	25
25	Hot waste gas	62.75	1	850
26	Cooled waste gas	62.75	0.1	350
27	Waste gas	62.75	0.1	50
28	Fe_2_O_3_	100	1	850

**Table 3:** Electricity results of the proposed process.

Equipment	Value (kW)
Crusher	−25
Pyrolysis reactor	−20
Pump	−1
Compressor-1	−8
Compressor-2	−7
Expander-1	15
Expander-2	29
Expander-3	20
Net electricity	3

**Table 4:** Gas products and oxygen carrier results of the proposed process.

Item	Fuel Reactor	Steam Reactor	Air Reactor
	kmol/h		
H_2_	0.0415994	0.0149086	0
CO	0.284967	0	0
CH_4_	2.29202 × 10^−6^	0	0
CO_2_	1.15597	0	0
Fe	0	0	0
FeO	1.25241	1.22259	0
Fe_2_O_3_	0	0.0149086	0.626205
O_2_	2.29196 × 10^−16^	1.00022 × 10^−17^	0.276666
N_2_	0	0	2.19061
H_2_O	0.212528	2.48297	0

**Table 5:** Properties of rice straw [24].

Proximate analysis (w_ad_/%)	
Ash	9.22
Volatile Matter	69.16
Fixed Carbon	14.86
Moisture	6.76
Ultimate analysis (w_daf_/%)	
C	44.50
O	45.66
H	6.24
N	1.45
S	2.15

**Table 6:** The simulation and experiment results of the rice straw pyrolysis [24].

Product		Simulation (wt% of Rice Straw)	Experiment (wt% of Rice Straw)	Relative Error (%) *
Pyrolysis gas	CO	17.01	18.68	8.94
	CO_2_	29.51	28.84	2.32
	H_2_	1.53	1.73	11.56
	CH_4_	3.66	3.83	4.44
	Others	1.04	0.92	13.04
Bio-oil		23.07	21	9.86
Biochar		24.18	25	3.28

* Relative error = (|Experiment–Simulation|/Experiment) × 100%.

**Table 7:** Aspen model and input data for the equipment of the proposed system.

Equipment	Aspen Model	Design Parameters
Crusher	Crusher	25 °C, 0.1 MPa
Separator	SSplit	600 °C, 0.1 MPa
Flash drum-1	Flash	600 °C, 0.1 MPa
Flash drum-2	Flash	25 °C, 0.1 MPa
Pyrolysis reactor	RYield, REquil and RGibbs	600 °C, 0.1 MPa
Fuel reactor	RGibbs	900 °C, 1 MPa
Steam reactor	RGibbs	700 °C, 1 MPa
Air reactor	RGibbs	980 °C, 1 MPa
Pump	Pump	25 °C, 1 MPa
Heat recovery steam generator	MHeatX	
Compressor-1	Compr	1 MPa
Compressor-2	Compr	1.2 MPa
Expander-1, Expander-2 and Expander-3	Compr	0.1 MPa

## Data Availability

The data were contained within the article.

## References

[1] Pashchenko D. (2022). Liquid organic hydrogen carriers (LOHCs) in the thermochemical waste heat recuperation systems: The energy and mass balances. Int. J. Hydrog. Energy.

[2] Pashchenko D., Mustafin R., Karpilov I. (2022). Thermochemical recuperation by steam methane reforming as an efficient alternative to steam injection in the gas turbines. Energy.

[3] Zhang J., Zhang T., Ma J., Wang Z., Liu J., Gong X. (2021). ORR and OER of Co-N codoped carbon-based electrocatalysts enhanced by boundary layer oxygen molecules transfer. Carbon.

[4] Zhou X., Zhao J., Guo S., Li J., Yu Z., Song S., Li J., Fang Y. (2018). High quality syngas production from pressurized K_2_CO_3_ catalytic coal gasification with in-situ CO_2_ capture. Int. J. Hydrog. Energy.

[5] Pashchenko D. (2019). Experimental investigation of reforming and flow characteristics of a steam methane reformer filled with nickel catalyst of various shapes. Energy Convers. Manag..

[6] Samprón I., de Diego L.F., García-Labiano F., Izquierdo M.T., Abad A., Adánez J. (2020). Biomass Chemical Looping Gasification of pine wood using a synthetic Fe_2_O_3_/Al_2_O_3_ oxygen carrier in a continuous unit. Bioresour. Technol..

[7] Zhou X., Li G., Liu F., Li N. (2022). Production of ethanol from corn straw based on chemical looping gasification: Economic analysis. Bioresour. Technol..

[8] Pashchenko D. (2019). Experimental investigation of synthesis gas production by methane reforming with flue gas over a NiO-Al_2_O_3_ catalyst: Reforming characteristics and pressure drop. Int. J. Hydrog. Energy.

[9] Xu D., Yang S., Su Y., Shi L., Zhang S., Xiong Y. (2021). Simultaneous production of aromatics-rich bio-oil and carbon nanomaterials from catalytic co-pyrolysis of biomass/plastic wastes and in-line catalytic upgrading of pyrolysis gas. Waste Manag..

[10] Kan T., Strezov V., Evans T.J. (2016). Lignocellulosic biomass pyrolysis: A review of product properties and effects of pyrolysis parameters. Renew. Sustain. Energy Rev..

[11] Zou J., Oladipo J., Fu S., Al-Rahbi A., Yang H., Wu C., Cai N., Williams P., Chen H. (2018). Hydrogen production from cellulose catalytic gasification on CeO_2_/Fe_2_O_3_ catalyst. Energy Convers. Manag..

[12] He C., Feng X., Chu K.H. (2013). Process modeling and thermodynamic analysis of Lurgi fixed-bed coal gasifier in an SNG plant. Appl. Energy.

[13] Liu L., Cao Y., Ma D., Liu Q., Yang J. (2017). Process simulation of coal-direct chemical looping gasification for syngas production. RSC Adv..

[14] Li F., Zeng L., Fan L. (2010). Biomass direct chemical looping process: Process simulation. Fuel.

[15] Zhou X., Yang X., Li J., Zhao J., Song S., Hao Z., Li C., Zhang J., Yu Z., Fang Y. (2020). High-pressure rapid hydrogasification of pinewood for methane production using calcium looping concept. Energy Convers. Manag..

[16] Ma S., Chen S., Zhu M., Zhao Z., Hu J., Wu M., Toan S., Xiang W. (2019). Enhanced sintering resistance of Fe_2_O_3_/CeO_2_ oxygen carrier for chemical looping hydrogen generation using core-shell structure. Int. J. Hydrog. Energy.

[17] Jia L., Cheng P., Yu Y., Chen S.H., Wang C.X., He L., Nie H.T., Wang J.C., Zhang J.C., Fan B.G. (2023). Regeneration mechanism of a novel high-performance biochar mercury adsorbent directionally modified by multimetal multilayer loading. J. Environ. Manag..

[18] Nguyen T., Clausen L.R. (2019). Techno-economic analysis of polygeneration systems based on catalytic hydropyrolysis for the production of bio-oil and fuels. Energy Convers. Manag..

[19] Situmorang Y.A., Zhao Z., An P., Yu T., Rizkiana J., Abudula A., Guan G. (2020). A novel system of biomass-based hydrogen production by combining steam bio-oil reforming and chemical looping process. Appl. Energy.

[20] Zhang Y., Lv P., Wang J., Wei J., Cao P., Bie N., Bai Y., Yu G. (2022). Product characteristics of rice straw pyrolysis at different temperature: Role of inherent alkali and alkaline earth metals with different occurrence forms. J. Energy Inst..

[21] Zhang L., Kong S. (2012). Multicomponent vaporization modeling of bio-oil and its mixtures with other fuels. Fuel.

[22] Zeng D., Cui D., Qiu Y., Li M., Ma L., Zhang S., Xiao R. (2020). Mn-Fe-Al-O mixed spinel oxides as oxygen carrier for chemical looping hydrogen production with CO_2_ capture. Fuel.

[23] Bock S., Stoppacher B., Malli K., Lammer M., Hacker V. (2021). Techno-economic analysis of fixed-bed chemical looping for decentralized, fuel-cell-grade hydrogen production coupled with a 3 MWth biogas digester. Energy Convers. Manag..

[24] Boughanmi H., Lazaar M., Farhat A., Guizani A. (2017). Evaluation of soil thermal potential under Tunisian climate using a new conic basket geothermal heat exchanger: Energy and exergy analysis. Appl. Therm. Eng..

[25] Dai B., Zhang L., Cui J., Hoadley A., Zhang L. (2017). Integration of pyrolysis and entrained-bed gasification for the production of chemicals from Victorian brown coal-Process simulation and exergy analysis. Fuel Process. Technol..

[26] Cruz P.L., Iribarren D., Dufour J. (2017). Exergy analysis of alternative configurations of a system coproducing synthetic fuels and electricity via biomass gasification, Fischer-Tropsch synthesis and a combined-cycle scheme. Fuel.

[27] Xiong J., Zhao H., Zheng C. (2011). Exergy Analysis of a 600 MWe Oxy-combustion Pulverized-Coal-Fired Power Plant. Energy Fuels.

[28] Abuadala A., Dincer I., Naterer G.F. (2010). Exergy analysis of hydrogen production from biomass gasification. Int. J. Hydrog. Energy.

[29] Tumen Ozdil N.F., Tantekin A., Erbay Z. (2016). Energy and exergy analyses of a fluidized bed coal combustor steam plant in textile industry. Fuel.

